# Altered Passive Eruption and Familial Trait: A Preliminary Investigation

**DOI:** 10.1155/2014/874092

**Published:** 2014-05-20

**Authors:** Roberto Rossi, Giorgio Brunelli, Vincenzo Piras, Andrea Pilloni

**Affiliations:** ^1^Department of Periodontology, University of Rome “Sapienza”, Italy; ^2^School of Dental Medicine, University of Cagliari, Italy

## Abstract

Altered passive eruption is described as a condition in which the relationship between teeth, alveolar bone, and the soft tissues creates an excessive gingival display and, in turn, in some circumstances, it may reveal a clinical aspect also known as the “gummy smile.” The surgical management of such cases is well understood and has been widely described, with mucogingival and osseous resective procedures being predictable surgical means leading to more balanced aesthetics and proper display of the teeth anatomy. The possible familial trait in case of passive eruption and therefore the possibility of recurrence of the same condition in families of siblings or parents of affected patients have been investigated in this study. 20 patients have been selected and treated in both a private practice and university settings and their immediate family trees were evaluated in order to understand the incidence of the condition. 65% of the treated patients had one or more family members with the same condition leading to seeking further investigation on the possible genetic correlation.

## 1. Introduction

The concept of “biologic width” is very important in all branches of dentistry, since a healthy interface between the teeth and the hard and soft tissues is the keystone in all successful restorative, periodontal, prosthetic, and orthodontic cases.

In 1996 Garber and Salama [1] published a paper that studied the biologic width on human cadaver teeth. They divided the dentogingival junction into the connective tissue attachment and the epithelial attachment. Their findings showed that the distance between the base of the epithelial attachment and the bone crest was constant and in all stages of eruption the average dimension was 1.07 mm. On the other hand the epithelial attachment was variable and the average dimension was 0.97 mm.

The same findings were shown in a study by Reinken and Van Oost in 1992 [2] investigating cadaver jaws describing that the connective tissue attachment is less variable than the epithelial attachment. Their results showed a mean dimension of 1.14 mm for the epithelial attachment and 0.77 mm for the connective tissue attachment. In 2004 Alpiste-Illueca [3] introduced a new method for measurement by using a reproducible radiographic technique. Their findings showed an average dimension of 2.05 mm for the distance between the cementum-enamel junction and the alveolar bone crest and 2.0 mm for the biologic width. Indeed, all the available literature shows that the dimensions of soft tissue attachment are highly variable and that such values could serve as a reference, but it also shows that measurements should be registered case by case given a certain range of variability.

## 2. Classification

The concept of delayed passive eruption was first introduced by Coslet et al. in 1977 [4]. Some patients with this condition in case of high lip line may present a smile with clinical crowns that appear short or shorter than they should be. The proposed classification evaluated the relationship between the gingiva and the clinical crown on one hand and the relationship between the CEJ and the bone crest on the other. Therefore their classification was subdivided as follows.

**Figure 1:**
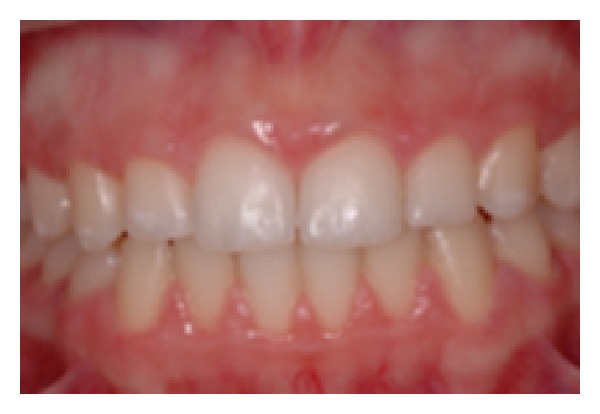
Aspect of Type I.

**Figure 2:**
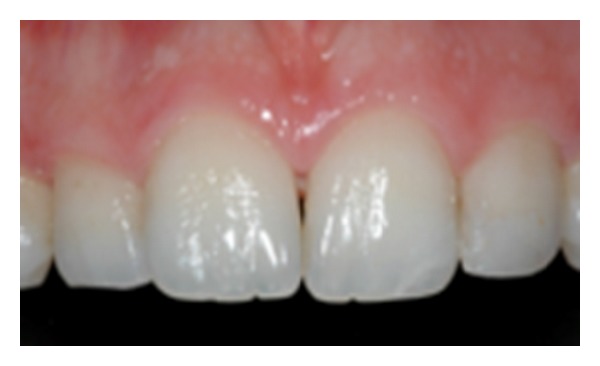
Aspect of Type II.

**Figure 3:**
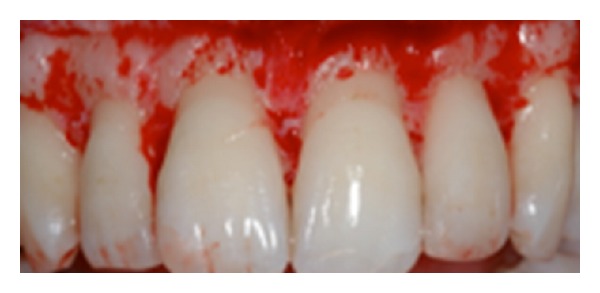
Normal biologic width in Subtype A.

**Figure 4:**
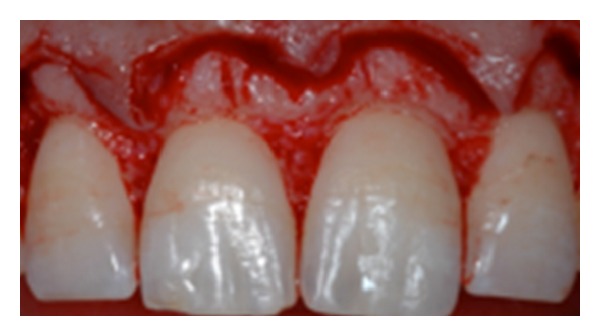
Bone crest at level of CEJ.

 Type  I: the gingival margin is incisal to the CEJ, the dimension of keratinized gingiva is wider than usual, and clinical crowns are short (Figure 1).
 Type  II: the dimension of the gingiva from the gingival margin to the mucogingival junction appears normal. The free gingival margin is incisal or occlusal to the CEJ and the mucogingival junction is positioned at the CEJ (Figure 2).
 Subtype  A: the distance of alveolar crest to CEJ is approximately 1.5 mm: in such cases a normal attachment can be found (Figure 3).
 Subtype  B: the alveolar crest is at the level of the CEJ or above (Figure 4).

In the past, there has been evidence of the impact of systemic control during mammalian growth by different hormones or growth factors that might be implicated and therefore explain their modulation during developmental events such as palate and skin differentiation, eye opening, and tooth eruption [5]. Based on the above-mentioned data our study wanted to understand whether or not patients with passive eruption have siblings or parents presenting with the same anatomical characteristics.

## 3. Materials and Methods

20 patients, 10 male and 10 female, with age range 18–45 entered the study and were treated between 2008 and 2012 in a private practice setting and in the Section of Periodontics of the University of Rome “Sapienza.” 18 years old were considered the youngest patients that could enter the study having completed their body growth at this age [2]. Patients and their immediate family (brothers, sisters, and parents) after signing an informed consent were invited to come to both clinics for checkup and evaluation of their condition. All patients evaluated, given the young age, had the whole family available for evaluation. Those whose family members did not show up for checkup were excluded from the evaluation.

**Figure 5:**
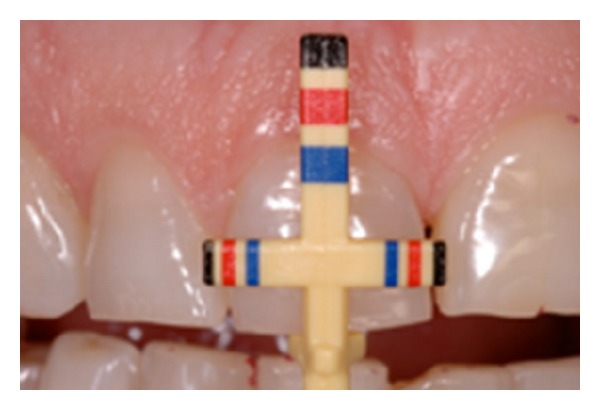
Chu's probe for esthetic proportions.

Treatment of patients showing altered passive eruption involves the initial evaluation and assessment of the smile “dynamics” and periapical radiographs were taken to measure and superimpose the clinical situation to the “hidden” anatomy. The clinical evaluation was also helped by the use of the Chu et al. probes [6, 7] instruments that help evaluate the proportions of the upper anterior teeth in relation to the esthetic and smile design (Figure 5).

As suggested by Garber and Salama in 1996 there are only two treatment options for cases of altered passive eruption: a simple gingivectomy to expose the hidden anatomy in cases of type 1-A and a full thickness flap with osseous resective surgery and an apically repositioned flap [1]. Patients were given oral hygiene instructions and received full mouth scaling and root planing before being treated either with gingivectomy or flap surgery. In all patients the length of teeth was measured clinically and radiographically at baseline to assess the amount of soft tissue resection, and presurgical and intrasurgical pictures assessing the position of the bone crest before and after the osseous resection were taken. Healing and remodelling of the soft tissue were evaluated at 1, 3, 6, 12 weeks and 6 months and one year after surgery, by means of intraoral pictures.

All patients were asked to invite their immediate family (parents and siblings) to the private practice or the university clinics for evaluation of their clinical conditions and to register if and how many within the family were showing signs of altered passive eruption (see Table 1).

**Figure 6:**
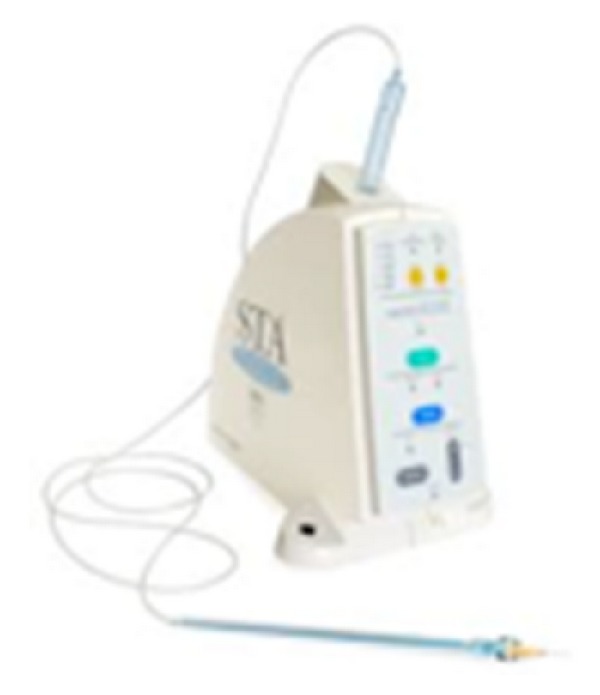
STA device.

**Figure 7:**
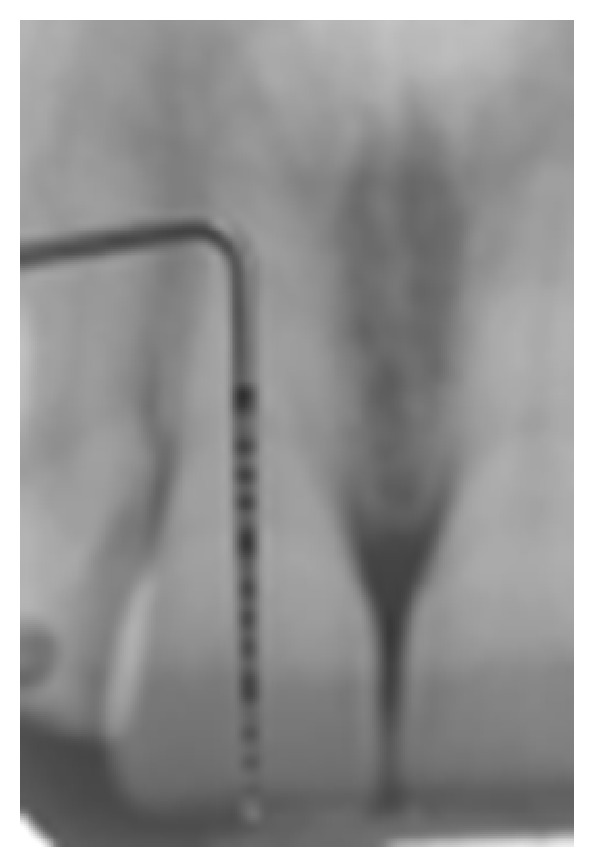
Anatomy of the tooth measured on periapical rx.

**Figure 8:**
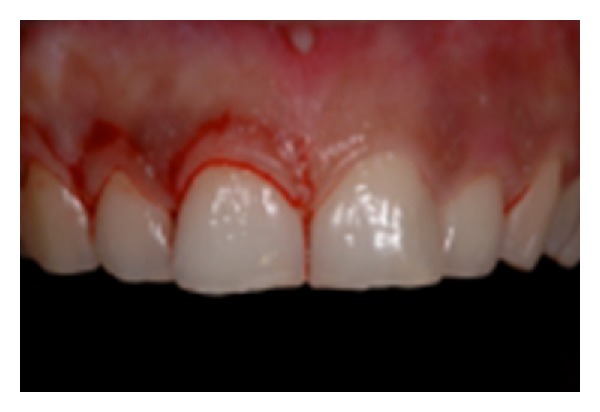
Flap design adapted to the hidden anatomy.

**Figure 9:**
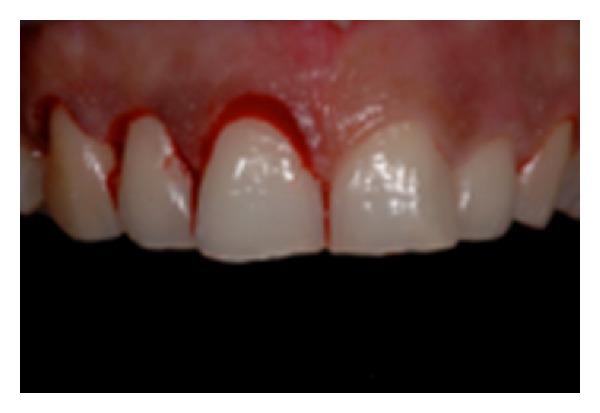
Real anatomy compared to altered passive eruption.

**Figure 10:**
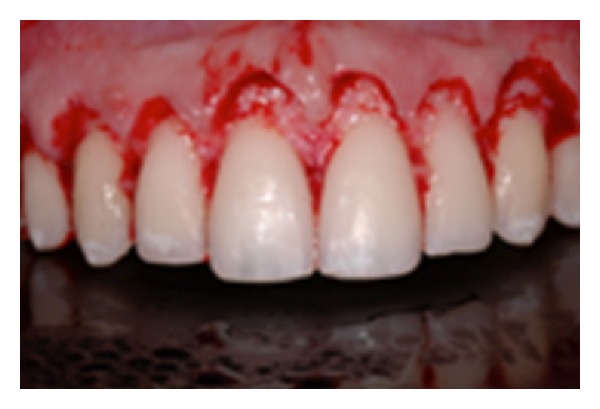
Flap elevation displays the condition.

*Surgical Procedure.* Anesthesia was administered with the use of an electronic controlled device (STA (single tooth anesthesia) Milestones Scientific, Livingstone, NJ, USA) (Figure 6). The microprocessor would administer anesthesia with the AMSA and PSA technique described by Friedman and Hochman [8, 9] and articaine 1 : 200.000. The use of this technique would allow all the patients to be anesthetised, but having the full control of the upper lips, in order to assess their smiles during the surgical procedure. Teeth were measured from the periapical X-rays using a PCP15 periodontal probe (Figure 7), and the flap design was done according to the anatomy and the amount of keratinized tissue (Figure 8). In all cases the soft tissue was removed first on the upper right side of the mouth to expose the original anatomy in comparison to the “gingival smile” (Figure 9). After that the soft tissue of the left side of the arch of the involved teeth was removed and the flap elevated to expose the underlying bone (Figure 10).

**Figure 11:**
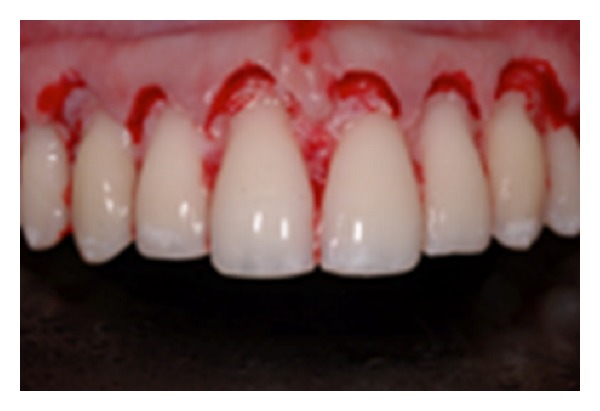
After osseous resective surgery.

**Figure 12:**
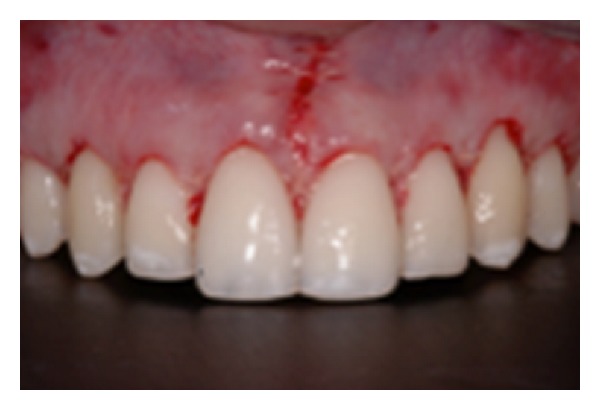
Flaps are sutured in apical position at CEJ.

**Figure 13:**
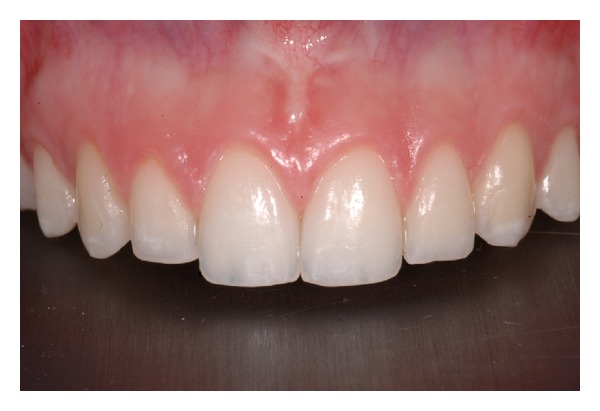
Uneventful healing with proper display of the anatomy.

At this point the assessment of the biologic width was registered and the osseous respective surgery was performed according to the protocols of Schluger 1949 [10]. After bone remodelling (Figure 11) soft tissues were sutured apical to the CEJ with single interrupted sutures (Figure 12). Patients were reevaluated at 7–14 days for suture removal and evaluation of the soft tissue response. (Figure 13).

## 4. Results

Of 20 patients evaluated, 13 had one of their siblings showing altered passive eruption of some form (65%), 6 had one or both of the parents presenting the condition (30%), 3 had the whole family group showing the condition (15%), and 6 patients (30%) had no family members presenting the condition. The results of this preliminary investigation show that there seems to be a positive correlation between family members and passive eruption. In this small group more than 50% of the evaluated patients had one family member showing the same clinical situation and 15% of the sample had all family members showing altered passive eruption.

It would be interesting to evaluate a larger group of patients to confirm the results of this preliminary investigation and, at the same time, have the opportunity to investigate whether even the type of passive eruption has a positive relationship within the family members.

## 5. Discussion

Altered passive eruption is well described in the literature and the surgical solutions are well known. From a strict perspective of analyzing the prevalence of such condition it would be important to investigate whether individuals showing this condition have one or more family members with the same problem [1, 3, 4, 8, 11–14].

In encountering several cases of passive eruption in the daily practice we decided to analyze surgically treated cases and their families in a five-year period of time. 20 families accepted to participate in the study. The results of this evaluation pointed out a strong correlation, since 65% of the patients had at least one family member showing the same condition, and 15% had the whole family group with altered passive eruption. No correlation has been investigated with respect to the type of passive eruption between family members.

It is important to note that there are no data in the periodontal literature to date reporting information on this matter and therefore this preliminary study should pose the basis for further investigations with a larger number of patients. With this small sample size one may speculate a high correlation with a familial trait since in only 6 of 20 patients there was no other member of the family presenting with such gingival appearance. Future studies should also investigate whether patients presenting altered passive eruption possess specific types or altered clinical manifestations of growth during their lives and, in this case, understand from the medical history whether or not external factors might have had an influence on the development of their individual phenotype.

## 6. Conclusions

Proper diagnosis of the smile type is crucial for a successful procedure that might increase chances for patients seeking to obtain and expose their “original” smile. This paper took into consideration the fact that patients showing this condition might have one or more family members with the same clinical aspect. Given the small size, further investigations with a larger number of patients would be necessary to confirm the hypothesis that altered passive eruption seems to be a condition that might carry some genetic trait and why it can often be seen among family members.

## Figures and Tables

**Table 1:** Qualitative evaluation of altered passive eruption in siblings of treated patients.

Patient ID	Age	Siblings	Father	Mother
A.P	38	Brother	n	n
G.P	42	Sister	n	n
C.P	25	Brother	n	n
T.P	21	Sister	n	n
T.O	32	n	n	n
S.S	41	Sister	n	n
L.S	45	Brother	n	n
U.Y	27	n	n	n
D.M	33	n	n	n
I.P	29	n	n	n
C.P	30	Sister	n	n
V.B	18	n	n	n
M.J	23	Sister	y	n
M.F	25	Brother	y	n
N.F	18	Brother	y	y
N.G	24	Sister	y	y
R.S	20	Sister	y	y
N.J	30	n	n	n
V.T	26	n	y	n
M.P	18	Brother	n	n
